# Glasgow Royal Infirmary

**Published:** 1831-03

**Authors:** 


### GLASGOW ROYAL INFIRMARY.

*Affections of the lower Extremities.* [Extract from Dr. Weir's Report, &c. in Glasgow Medical Journal.]

Case I. *Injury of Ankle-joint; Purulent Deposition in opposite Knee.*

R. W. aged sixty, admitted 14th January. About six weeks before admission, fell down a coal-pit, and struck right leg against the point of a pick, which entered the ankle-joint, and by report passed completely through it. The hemorrhage from the wound was profuse, and continued occasionally for fourteen days. He also, at same time, suffered a simple fracture of upper third of tibia of opposite limb. On admission, the lower half of right leg was greatly swollen, the integuments undermined for a considerable extent upwards, and there was an opening on each side of the ankle-joint. From the opening at the external malleolus the probe passed easily into the joint, and the tarsal bones were felt rough; from that on the opposite side the probe passed upwards for several inches along the tibia. From both these openings there was a discharge

*urethra into a female one*, and that he, at various times, performed the operation on the dead subject, by introducing into the bladder the dilator, from an incision made into the membranous portion of the urethra, through the prostatic portion, and always extracted the stone with facility. Once only did he use the dilator on the living subject: it was in a case very similar to that of Robert Brock, alluded to by Dr. Buchanan; and on this occasion, two or three calculi, which had become impacted in the urethra, were extracted: one of the calculi, which was thrown back into the bladder, was easily removed after the use of the dilator, as witnessed by Mr. Walker, now assistant surgeon, and at that time a pupil, at the hospital. On consideration, however, Mr. Keate did not deem it expedient to recommend its adoption on the living subject, partly from its unnecessarily protracting the operation, partly from experience of the comparative safety of the usual mode in a healthy subject and a healthy bladder; while it seemed likely to increase the dangers, or at all events not to lessen them, in cases of diseased prostate or bladder; when, and perhaps when alone, a substitute for the present mode of operating would certainly be desirable.

Mr. Keate does not mean that his observations should be construed into any wish, on his part, to detract from the merits or originality of Dr. Buchanan's suggestions; for he thinks it probable that Dr. B. might never have heard of his experiments, which were made in the presence only of one of the surgeons, the house-surgeon, and a pupil or two, in the dead-house of St. George's Hospital, some seven or eight years ago.

Mr. Keate says, in reference to the subject of *lithotritie*, that it would be a much more valuable and interesting operation, if it were adapted to cases of diseased prostate, or of large stones, and to children; but, as far as he had seen or formed any judgment, it appeared only to be applicable to those cases of moderate-sized stone, healthy constitutions, and sound prostates, in which the common lateral operation is, under usual circumstances, capable of being quickly and safely performed, with, he thinks, at least, as little suffering as is caused by lithotrity, and without the necessity for painful repetition, and the chances of leaving a nucleus for future urinary depositions; one instance of which had lately come within his own knowledge.—Editor.

of fœtid purulent matter in considerable quantity. General health much fallen off. Countenance pale and sunk. Pulse 120, very feeble. Complains of pain at lower part of chest, increased on inspiration; has also considerable cough, but no expectoration. Habits intemperate. No treatment, but poultices to limb. Fracture of left tibia has united.

An opening was made a few inches above inner ankle, and four ounces of very fœtid pus let out. Through this the lower end of tibia was felt bare. He was ordered eight ounces of wine daily, and a grain of opium with two of calomel morning and evening. On the 16th, two days after admission, he had recruited a little, and it was thought that, notwithstanding his pneumonic symptoms, he had some chance of recovery by removing the leg. Amputation was accordingly performed a few inches below knee. The cartilages of tibia and astragalus were found completely destroyed, and the extremity of fibula was so soft as to be easily cut with the scalpel. The os calcis, the naviculare and cuboides, were in a state of caries, and the disease had also affected the ends of the metatarsal bones to some extent. There was nearly an ounce of fœtid pus under the astragalus.

For some days after the operation he recruited very considerably: he slept well, his cough was much relieved, and his appetite had somewhat returned. His pulse, however, never fell below 110; his countenance remained anxious, and his breathing oppressed. He was allowed wine freely, and occasionally some spirits, having been accustomed to drink very much for many years. On the 20th he had some diarrhœa, but was otherwise improving. The stump was found to have adhered in the centre, and the discharge was moderate. By next dressing, on the 22d, the wound was somewhat sloughy; the diarrhœa continued, notwithstanding astringents and opium; the pulse was 130, and he was evidently sinking. Next day he was in the following state: "Had a rigor last night, which lasted fifteen minutes. Slept ill. Cough much more severe, and expectoration tinged with blood. Was occasionally incoherent during the night. Pulse 150, feeble. Countenance sunk." The following night had another severe rigor, and died on the evening of the 24th, eight days from the operation.

*Inspection.* Stump wholly open and sloughy, but knee-joint quite healthy. Left knee-joint contained an ounce of thick greenish fœtid pus. Synovial membrane much thickened, inflamed, and in a state of ulceration. Lungs appeared very prominent, and completely occupying the cavity of the thorax; they were of a very dark colour, and œdematous, and there was an extensive effusion of a black coloured fluid into their cellular texture. The usual crepitating sound was wanting, and many small tubercles were interspersed throughout their substance. There were no adhesions, and no effusion into the cavity of the pleura. A small portion of the lung put into water tinged the fluid of a deep black colour.

I consider death to have been produced in this case in consequence of the affection of the lungs, along with the deposit of pus in the knee-joint, occurring in a constitution previously debilitated by intemperance and a long-continued local irritation and discharge.

Case II. *Caries of Tibia, with Ulceration of Cartilages of Knee-joint; Purulent Deposition in opposite Knee.*

J. G., aged thirty-six, previously healthy, was seized, about the beginning of December, with acute pain and swelling of upper part of left leg, accompanied with smart symptomatic fever. Leeches, cold applications, blisters, and other means, were employed for four weeks; but suppuration took place, and an incision was made over upper part of tibia, and a large quantity of purulent matter discharged; afterwards another opening was made on fibular side of leg. On his admission into the hospital on 12th January, the knee-joint measured one-third larger than the opposite. The integuments covering it were of a red colour, and it was acutely painful on pressure. There were two sinous openings immediately below joint, one on each side, which discharged a very considerable quantity of thin curdy matter. General health much impaired. Appears feeble and emaciated; countenance sallow; pulse 100, small; bowels loose; no appetite.

On admission, the openings were enlarged. He was ordered eight ounces of wine daily, two grains of quinia night and morning, and a grain of opium twice a day. The disease which appeared to be going on in the knee, increased rapidly. The joint became more swollen, and in a day or two the probe could be passed, from the sinus over tibia, into the joint. The discharge continued copious, the diarrhœa rather increased, and his general health did not improve. On the 19th, seven days after admission, he was in the following state: "Strength has been gradually falling off since admission; pain of knee-joint and leg has been more severe, and the discharge more copious and unhealthy; pulse 120, more feeble; appetite bad, and bowels still loose." The limb was now removed at mid-thigh, by the double-flap operation. On inspecting the leg, the head of the tibia was found deprived of cartilage, and in a state of caries. The upper part of the shaft was also carious, and numerous foramina of small size were visible on its surface. On the posterior part of the bone new osseous particles, or bony granulations as they may be called, were thrown out in great abundance, while a small portion on its fibular side presented the appearance of incipient necrosis. The cartilage covering the head of femur was somewhat thickened. About an ounce of dark-coloured fœtid purulent matter was found within the joint.

This man, when the source of irritation was removed, improved for two or three days; but, on the third day after the operation, he became slightly incoherent, and his pulse got up to 120. Next day he had low muttering delirium: and on the 5th, he complained of pain in the opposite knee-joint, which appeared considerably swollen, but without tension or redness. He was treated with the carbonate of ammonia, opium, and a liberal allowance of wine, and a blister was applied to the knee. He continued, however, to get worse, and died with symptoms of low typhus, ten days after the operation. Besides the pain of knee, he complained also of pain in left wrist-joint, which, however, was not swollen.

On inspection, the stump was found wholly open. The soft parts, to the extent of four inches, were sloughy, separated from the bone, and covered with fœtid grayish-coloured matter. The bone was healthy. In the right knee-joint, there were about two ounces of very fœtid pus. The synovial membrane was considerably thickened, and in a few points inflamed: the cartilages of femur and tibia were partially destroyed; a small abscess had formed exterior to left wrist-joint. The other joints were examined, but found natural. There was no effusion into the pleura, and only some trifling adhesions existed. Both lungs were much loaded with bloody serum, and in the upper lobe of the right there was some slight degree of unnatural firmness, scarcely amounting to hepatization.

In these two cases, the appearances on dissection were, in many respects, similar. In both, there was a collection of purulent matter in the knee-joint, and some affection of the lungs. In the first, the disease in the lungs had gone to a great length; in the other, it was very slight, and by no means sufficient to account for death. There is this difference, however, in the two cases, that in the one there were evident symptoms, before death, of disease in the joint; there was acute pain and swelling; while, in the other, there was no symptom whatever to lead to a suspicion that disease was going on in that quarter.

These purulent depositions appear to occur more frequently after secondary, than after primary, amputations, and in general during the suppurating state of the wound. In secondary amputations, where there has been for many days a great discharge of pus, as in compound fractures, wounds of joints, &c. the constitutional symptoms, although they very generally abate a little after the removal of the local irritation, in a few days become more severe, and a sudden determination to some particular part takes place, to the lungs, the liver, or the joints; purulent depositions occur, and death soon follows. This does not appear to take place so often after primary amputations. The affection seems to arise from the system not being able to accommodate itself to the sudden change resulting from the want of the drain, to which it had been for some time habituated. "For although the continuance of the discharge would very soon have destroyed the patient, still he is not able to bear the sudden change on the removal of the limb; because the quantity of blood sent to it for the formation of pus, and the natural supply of the limb, was much greater in proportion to the quantity in circulation, than in a state of health."

Dr. Weir relates another case, in which, after extirpation of a scirrhous mamma, purulent depositions were found, upon examination after death, in both knee-joints, and in the frontal sinus: he correctly states, that, although "purulent depositions, after operations and injuries, have been found in the cavity of the pleura and abdomen, in the substance of the thoracic and abdominal viscera, in the cellular substance, in various parts of the body, in the knee-joint and almost every other articulation, and even within the cranium: their occurrence in the frontal sinuses, or other cavities lined with a perfect mucous membrane, appears to be very rare."

